# Retracted: Nursing Observation on the Clinical Efficacy and Toxicity of Lobaplatin Compared with Cisplatin in the Treatment of Locally Advanced Hypopharyngeal Carcinoma Based on Intelligent CT Imaging

**DOI:** 10.1155/2023/9846052

**Published:** 2023-01-19

**Authors:** Journal of Healthcare Engineering

*Journal of Healthcare Engineering* has retracted the article titled “Nursing Observation on the Clinical Efficacy and Toxicity of Lobaplatin Compared with Cisplatin in the Treatment of Locally Advanced Hypopharyngeal Carcinoma Based on Intelligent CT Imaging” [1] due to concerns that the peer review process has been compromised.

Following an investigation conducted by the Hindawi Research Integrity team [2], significant concerns were identified with the peer reviewers assigned to this article; the investigation has concluded that the peer review process was compromised. We therefore can no longer trust the peer review process, and the article is being retracted with the agreement of the Chief Editor.

We were unable to contact the corresponding author on the email address provided to us.
